# Pubertal timing and tempo differentially influence cortical and subcortical maturation in adolescence

**DOI:** 10.1016/j.dcn.2025.101657

**Published:** 2025-12-12

**Authors:** Clare F. McCann, Theresa W. Cheng, Kathryn L. Mills, Jennifer A. Silvers

**Affiliations:** a University of California, Los Angeles, Los Angeles, CA, USA; b Department of Psychology, Harvard University, MA, USA; c Department of Psychology, University of Oregon, OR, USA

**Keywords:** Pubertal timing, Pubertal tempo, Structural brain development, Adolescence, ABCD Study®

## Abstract

Puberty is a developmental period marked by an influx of sex steroids, which trigger physical and psychological changes. Furthermore, puberty elicits changes in structural brain development that are distinct from those associated with chronological age. Emerging evidence suggests that interindividual differences in pubertal development, such as timing, whether one reaches puberty milestones before or after peers, and tempo, or whether one progresses through puberty at a slower or faster rate than peers, may also play a significant role in shaping structural brain development. The present study leverages longitudinal data from the **Adolescent Brain Cognitive Development**^**SM**^**(ABCD Study®)** to disentangle the influences of pubertal timing and tempo from chronological age on cortical and subcortical structural brain development during the adolescent period. Individuals with earlier timing tend to exhibit accelerated normative developmental trajectories compared to later timing peers, while individuals with faster tempos tend to exhibit thicker cortices, more cortical surface area, and greater subcortical volume compared to slower tempo peers. These findings underscore the significance of incorporating pubertal timing and tempo into models of structural brain development during puberty, thereby enhancing our understanding of variations in neurodevelopmental trajectories during adolescence.

## Highlights

• Individuals with earlier timing tend to exhibit accelerated normative developmental trajectories.
• Individuals with faster tempos exhibit thicker cortices, greater surface area, and subcortical volume than slower tempos.
• Pubertal timing and tempo act as distinct yet complementary predictors of adolescent structural neural trajectories.

## 1 Introduction

Puberty — a developmental process that co-occurs with the broader transition from childhood to adulthood, also known as adolescence — is much more than just an influx of sex steroids and the onset of reproductive maturation. Human and non-human animal research suggests that puberty is associated with structural reorganization (e.g., thickness, surface area, volume) of brain regions implicated in key socioemotional processes (e.g., emotion regulation, processing rewards) during adolescence (Herting and Sowell, 2017, Vijayakumar et al., 2018). Importantly, a growing body of evidence suggests that puberty elicits distinct changes in structural brain development from chronological age, despite age and puberty being highly correlated (Curtis et al., 2024, Vijayakumar et al., 2021, Vijayakumar et al., 2021, Wierenga et al., 2018). While much research has demonstrated that pubertal stage or hormone levels are associated with various features of brain development, emerging findings suggest that other aspects of puberty such as whether one is reaching milestones of puberty before or after peers (timing) and whether one progresses through puberty slower or faster than peers (tempo) may also play a significant role in dictating structural brain development. The present study leverages longitudinal data from the **Adolescent Brain Cognitive Development**^**SM**^ **(ABCD Study®;** Volkow et al., 2018**) Study** to disentangle the influences of pubertal timing and tempo from chronological age on cortical and subcortical structural brain development during the adolescent period.

### 1.1 Age-related structural maturation

Chronological age has been broadly associated with reductions in cortical thickness and volume, and a quadratic-like (slight increase to plateau to slight decrease) pattern of development in cortical surface area, during adolescence (Bethlehem et al., 2022, Mills et al., 2014, Vijayakumar et al., 2016). However, longitudinal data, including in the ABCD Study (Bottenhorn et al., 2023), have revealed marked variability in within-individual trajectories of brain development. Variability in pubertal development (absolute pubertal stage as well as relative timing and tempo) appears to explain in part why structural and functional neurodevelopment do not always align perfectly with chronological age (e.g., Dai and Scherf, 2019; Goddings, 2015; Herting and Sowell, 2017; Vijayakumar et al., 2018).

### 1.2 Pubertal development and structural maturation

Most research examining links between puberty and brain development to date has sought to identify associations between pubertal stage and brain development, while controlling for age (i.e., comparing brain development for individuals of the same age at different stages of puberty; e.g., Byrne et al., 2023; Curtis et al., 2024; Goddings et al., 2014; Wierenga et al., 2018). While such approaches can grant insights into how being generally more or less advanced in pubertal development than peers relates to neurodevelopment, they fail to provide insight into how the age at a specific puberty milestone (i.e., when an individual reaches Tanner Stage 3) relates to brain development. This methodological choice has been partially driven by necessity, given that much prior work on puberty has been cross-sectional or in sample sizes too small to distinguish between pubertal timing and tempo statistically. However, the emergence of large-scale longitudinal studies, such as ABCD, allows for novel interrogations of puberty, including more straightforward operationalizations of pubertal timing and tempo relative to peers and brain development.

*Pubertal Timing*. Research suggests that while increasing age is generally associated with reductions in cortical thickness and volume during adolescence, earlier pubertal timing accelerates these neurodevelopmental changes. Recent research from the first two time points of the ABCD Study finds that earlier pubertal timing is associated with accelerated structural development across multiple indices, including cortical thinning, reductions in cortical volume, and greater BrainAGE (a composite index of brain maturation) (Dehestani et al., 2023, MacSweeney et al., 2023, Whitmore et al., 2023, Wiglesworth et al., 2023). Earlier pubertal timing at one time point has been related to reduced cortical thickness and volume in temporal, frontal, and parietal regions compared to same-aged, same-sex peers at a later time point (MacSweeney et al., 2023, Wiglesworth et al., 2023). The effects were stronger in females, and those with earlier pubertal timing displayed thinner cortices on average (MacSweeney et al., 2023, Wiglesworth et al., 2023). At the subcortical level, earlier pubertal timing at one time point has been related to increased volume of the ventral diencephalon in males (MacSweeney et al., 2023) and reductions in volume in the thalamus, caudate, and accumbens in males and females (Ji et al., 2024), but no differences in amygdala volume (Thijssen et al., 2020, Thijssen et al., 2022) in males and females at a later time point.

*Pubertal Tempo*. Few investigations of the effects of pubertal tempo on structural brain development have been published. One study found that a faster tempo is related to accelerated development in various regions of the prefrontal and parietal cortices and hippocampus in males (Vijayakumar et al., 2021, Vijayakumar et al., 2021). Others find that the rate of change in pubertal development was related to less thinning in the superior frontal cortex and weakly related to the rate of change in a composite of neural maturation, with those exhibiting faster pubertal tempo also showing accelerated rates of a brain development (Herting et al., 2015, Holm et al., 2023).

While inconsistent, these results suggest that pubertal tempo may be a potential indicator of variability in structural brain development. Moreover, given that both timing and tempo explain variance in psychosocial wellbeing in adolescence (Cheng et al., 2020, Pfeifer and Allen, 2020), including mood symptoms (Keenan et al., 2014), externalizing behaviors (Marceau et al., 2011), and peer relationships, more research is needed to unpack how both of these features of pubertal development impact the developing brain.

### 1.3 Present study

The present study aims to investigate further the influence of pubertal timing and reveal the effects of tempo on structural brain development. We use five time points to model pubertal timing and tempo using logistic growth curve models that capture the characteristic S-shaped trajectory of pubertal development across age, rather than relying on linear mixed-effects models, which may inadequately represent the nonlinear nature of pubertal development. These models are based on meaningful developmental milestones, such as deviations from average patterns around mid-puberty, which provide more interpretable effects of pubertal timing and tempo. Building on prior studies using the first two time points of ABCD Study data, we will leverage *three* time points of surface-based morphometry neuroimaging data to investigate how pubertal timing and tempo influence variations in cortical thickness, surface area, and subcortical volume during adolescence. Specifically, we will use flexible nonlinear models to predict regional whole-brain structural development in males and females, rather than selecting specific regions of interest or using a global metric, to elucidate how these effects differ across hemispheres and regions. Understanding these relationships will provide critical insights into neurodevelopmental processes.

## 2 Methods

### 2.1 Study

The present paper leverages the longitudinal **Adolescent Brain Cognitive Development**^**SM**^ **(ABCD) Study**, which includes ∼11,800 adolescents (aged 9–10 at baseline). The ABCD Study is based in the United States and is a national ongoing longitudinal investigation of adolescent health, with a focus on assessing risk and resiliency factors linked to substance use (Karcher and Barch, 2021). For the purpose of the present paper, we use the three time points from Release 5.1 (https://nda.nih.gov/study.html?id=2313) to investigate our focal research questions using brain as the outcome and the full five time points to extract pubertal timing and tempo, all collected within the same window of time (the brain data were only collected at three out of the five time points: baseline, follow-up year 2, follow-up year 4). The final sample for the present project contained 6020 adolescents (F = 2803). ∼5848 adolescents were removed due to low-quality or missing neuroimaging, having less than 3 time points of data (for power purposes in the logistic growth curve model to extract individual pubertal development trajectories), belonging to a duplicate family ID, being scanned on a scanner with too few observations for harmonization, having more than one instance of “backwards” puberty (discussed further below) or identifying as intersex (sample too small to detect meaningful differences). See Supplementary Material Fig. 1 for a flowchart outlining the exclusion criteria for the present study. Participants excluded from the present project differed significantly from those included in our study in terms of race (Black and Hispanic youth were more likely to be excluded). We randomly selected only one participant from each family ID to control for complex family-related factors. The larger ABCD Study excluded initial participants based on the following criteria: non-English proficiency, general MRI contraindications, a history of a major neurological disorder, traumatic brain injury, extreme premature birth (<28 weeks gestational age), a diagnosis of schizophrenia, intellectual disability, moderate to severe autism spectrum disorder, or substance abuse disorder (Karcher and Barch, 2021). All participants completed an assent, and their caregivers completed a consent form before participation.

**Fig. 1:**
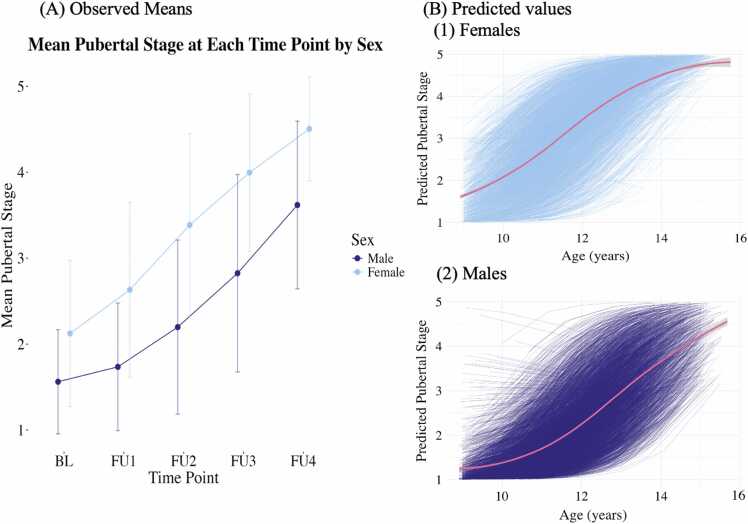
Visualization of Pubertal Development. Panel A: Mean of the Tanner-Stages from the converted Pubertal Development Scale at each time point. Panel B: Predicted trends based on growth curve models of pubertal stage across observations for females (1) and males (2). Note: Participants with predicted negative trajectories were removed from subsequent analyses. FU = follow-up to number relative to baseline.

### 2.2 Participants

In our final sample, 62.7 % of our participants identified as non-Latine White (see Table 1 for the full racial breakdown). 12.6 % reported an annual household income below $35,000 (see Table 1 for the full income-to-needs ratio breakdown).

**Table 1:** Sample characteristics by sex.

	**Males (n = 3217)**	**Females (n = 2803)**
*Age (mean years, standard deviation)*		
age at baseline	9.93 (0.62)	9.91 (0.6)
age at FU1	10.94 (0.63)	10.91 (0.62)
age at FU2	12.01 (0.65)	11.99 (0.65)
age at FU3	12.92 (0.64)	12.89 (0.63)
age at FU4	14.06 (0.66)	14.05 (0.65)
*Racial & Ethnic Identity*		
White	2005 (62.3 %)	1722 (61.4 %)
Black or African American	276 (8.6 %)	302 (10.8 %)
Native American	7 (0.2 %)	9 (0.3 %)
Pacific Islander Native	3 (0.1 %)	1 (0 %)
Indian	24 (0.7 %)	11 (0.4 %)
Chinese	40 (1.2 %)	23 (0.8 %)
Korean	8 (0.2 %)	11 (0.4 %)
Filipino	14 (0.4 %)	16 (0.6 %)
Japanese	7 (0.2 %)	10 (0.4 %)
Vietnamese	1 (0 %)	7 (0.2 %)
Asian (not further specified)	6 (0.2 %)	7 (0.2 %)
Latine	22 (0.7 %)	33 (1.2 %)
Multiracial	804 (24.9 %)	651 (23.2 %)
*Income-to-needs Ratio*		
Income-to-needs at baseline	4.43 (2.33)	4.47 (2.32)

*Note*. FU = follow-up to number relative to baseline; Higher values for the income-to-needs ratio indicate that individuals have more financial resources relative to their basic needs.

### 2.3 Measures

#### 2.3.1 Sex

Parent-reported child assigned sex at birth was used to administer the sex-specific pubertal measure described below and to separate the sample into male and female to account for sex differences in the timing of puberty.

#### 2.3.2 Pubertal development

Pubertal status was assessed using the 5-item Pubertal Development Scale (PDS; Petersen et al., 1988) to examine the perceived development of secondary sex characteristics, including growth spurts, body hair growth, skin changes, breast development, and menarche in females, as well as voice changes and testicular growth in males. Items are rated on a 4-point scale (1 = no development; 2 = development has barely begun; 3 = development is definitely underway; and 4 = development is complete), except menstruation, which is coded 1 = has not begun, 4 = has begun. Thus, higher scores reflect more advanced pubertal development. We converted the categorical summary PDS scores to continuous Tanner-like Stages using a validated conversion method that correlates strongly with other metrics of pubertal development, such as hormones and clinician exams (Shirtcliff et al., 2009). See Fig. 1A for the means of pubertal stage at each time point.

25.93 % of female and 45.8 % of male participants exhibited at least one regressing case of puberty (e.g., moving from Tanner Stage 3 to Tanner Stage 2 between time point 1 and time point 2), which is not uncommon in a report method of assessing pubertal status. In our data, the majority of regression in the present sample occurred at earlier time points and differed by report item. Rates of regression were highest for the growth spurt report item, followed by body hair growth in males and skin changes in females. Regression rates have been observed in other studies, such as the Human Connectome Project (Omary et al., 2025), while not as extreme, the present sample contains more time points (more room for regression among certain items; e.g., regression rates among the skin changes item increased slightly at later time points). We maintained these cases to account for normal measurement noise, but ran a sensitivity analysis excluding participants with more than one instance of regression (60 females, 150 males).

#### 2.3.3 Age

The self-reported age at each time point was reported in months, transformed into years, and then standardized.

#### 2.3.4 Brain

The ABCD Study was collected using a harmonized protocol across three scanner manufacturers: a 3-T Siemens Prisma, a General Electric 750 scanner, or a Philips scanner. Participants watched a movie while T1-weighted data were acquired using magnetization-prepared rapid acquisition gradient echo scans with a 1 × 1 × 1 mm^3^ resolution. The present paper utilizes curated data; therefore, the processing details adhere to the standard ABCD procedure. Briefly, data were checked for adherence to the intended imaging parameters and underwent standard semi-automated structural processing with FreeSurfer version 5.3.0. The data was corrected for gradient nonlinearity distortions (e.g., applied estimated B_1_ bias fields). Images were then registered and remapped to an average reference brain in standard space. All data were visually reviewed for motion by trained technicians and recommended for exclusion based on imaging artifacts or irregularities.

Further processing procedures are detailed in Hagler et al. (2019). The present project examined cortical thickness, surface area, and subcortical volume using Freesurfer v5.3.0 atlases. The *Desikan-Killiany* atlas (Desikan et al., 2006; Fischl et al., 2004) includes 68 segmentations and was used to extract cortical thickness and surface area. Cortical thickness is quantified as the distance between the innermost and outermost boundaries of the cerebral cortex. On average, the degree of cortical thickness in the human brain ranges between 1 and 4.5 mm, with an average overall cortical thickness of ∼2.5 mm and significant differences between regions (Fischl and Dale, 2000). Cortical surface area, an indicator of cortical expansion, ranges from 600 to 7000 mm² in a region (Potvin et al., 2017). The *Aseg* atlas (Fischl et al., 2002) comprises 36 segmentations and was used to extract volumes of subcortical structures, ventricular spaces, cerebrospinal fluid (CSF), white matter, cerebral cortex, and cerebellar cortex. Subcortical volume refers to the size of regions located beneath the cerebral cortex (e.g., amygdala, hippocampus, brain stem), with values typically ranging from 500 to 150,000 mm³ (Potvin et al., 2016) in human adults.

#### 2.3.5 Covariates

We controlled for site ID as dummy-coded contrasts, as the ABCD Study is collected at 21 different research sites. We chose to control for, rather than include these variables as nested within our models, because they are features of data collection that may explain variability in the outcome but are not of theoretical interest (McCormick et al., 2023). We conducted sensitivity analyses to determine whether controlling for body mass index (BMI) and the income-to-needs ratio alters the original results, and we report these results in the Supplementary Materials. BMI and income-to-needs ratio were included as they are both previously established predictors of various features of neural development in adolescence (e.g., Hamer and Batty, 2019, Noble et al., 2015, Cui et al., 2023).

### 2.4 Analysis

#### 2.4.1 Longitudinal scanner harmonization

To mitigate potential scanner-related variability as ABCD data is collected across multiple sites, we implemented *longCombat* (version 0.0.0.90000). According to Beer et al. (2020), brain data should be harmonized with models as closely as possible to the actual model being tested. Therefore, the data was harmonized for each metric, sex, and model (1, 2a or b, 3a or b). As our focal models employ a generalized additive mixed model (GAMM) framework, and *longCombat* utilizes linear mixed modeling, we imposed similar “smooth” terms from GAMM in our *longCombat* models. Two scanners for females (scanners 7 and 8) and three scanners for males (scanners 4, 7, and 8) participants had to be removed as there were too few observations (<100) for *longCombat* to stabilize among the other scanners' effects. This did not result in the loss of any participants completely, only observations. Scanner 4 for males could not be estimated and scanners 7 and 8 were unreliably estimated (effects were not successfully being removed). Based on differences in effect sizes and the predictive utility before and after implementation, *longCombat* seemed to stabilize the individual scanner effects successfully. See the present OSF project (https://osf.io/mgjyh/files/xpbaf) for details on a visualization of the difference in scanner effects before and after harmonization for the final sample.

#### 2.4.2 Pubertal timing and tempo

Prior research has indicated that the brain undergoes dynamic structural change during mid-puberty (Bottenhorn et al., 2023, Vijayakumar et al., 2018). The present study investigated how early or late an individual reaches Tanner stage 3, a marker of pubertal timing relative to peers, and how fast they progress, and whether this relative timing and tempo predicts differences in structural brain development.

A logistic growth curve model was used to derive pubertal timing and tempo in line with previously reported methods (Marceau et al., 2011). Fig. 1B visualizes individual trends in pubertal development. Because pubertal stages unfold from 1 to 5, a logistic growth curve model allows us to account for the natural lower (1) and upper (5) asymptotes. Further, a logistic growth curve model is recommended if data collection points do not completely capture puberty, which was possible in the present sample (McCormick et al., 2023). For example, a logistic growth curve model accounts for individuals who have already passed Tanner Stage 1 at the first time point or have not reached Tanner Stage 5 at the last time point. We compared the logistic model fit to the fits of linear, quadratic, cubic, and Gompertz curve models regressing pubertal status on age, separately for males and females.

The logistic growth curve model outperformed the linear, quadratic, cubic, and Gompertz curve models, as indicated by the Akaike Information Criterion, Bayesian Information Criterion, as well as out-of-sample performance and variance explained (see Supplementary Materials for the model fit values from each model). As mentioned earlier in the methods section, we ran a sensitivity analysis excluding participants with more than one instance of regression. The logistic growth curve model emerged as the best-fitting model again, and the fixed estimates remained the same. However, the overall model's fit improved when derived from the dataset excluding those with more than one instance of regression. Therefore, we removed these participants from subsequent analyses.

Timing was derived as an individual’s deviation from the estimated fixed effect of age at Tanner Stage 3 (12.99 in males, 11.36 in females). Estimated pubertal timing (or age at Tanner Stage 3) values ranged from 9.14 to 15.36 years in males and 7.97–14.94 years in females. For the deviation score, more positive timing values indicate later timing (i.e., an individual’s estimated age at Tanner Stage 3 was above the estimated fixed age at Tanner Stage 3 for the sample).

Tempo was derived as an individual’s deviation from the estimated speed of progression around the estimated age at Tanner Stage 3 (b = 0.96 in males, b = 0.95 in females). Eighteen males and eleven females were estimated to have negative alphas, which may reflect individuals with cases of regression or who were not stably estimated, and were therefore removed. Estimated pubertal tempo values ranged from 0 to 1.62 in males and 0–1.63 in females. More positive tempo values indicate a faster tempo (individual alphas are higher than the estimated fixed alpha).

In females, pubertal timing and tempo were moderately positively correlated (r = 0.35), indicating that those with later pubertal timing tend to have a faster tempo, which could reflect compensatory effects. In males, pubertal timing and tempo were moderately negatively correlated (r = -0.32), indicating that those with earlier pubertal timing tend to have a faster tempo. This finding is consistent with previous reports by Marceau et al. (2011) and Mendle et al. (2010).

Prior research within the ABCD Study suggests that younger participants respond less reliably using the PDS (e.g., Herting et al., 2020). However, they may become more reliable over time (as more information is gained about pubertal development). In our own analysis of the data, we found that parent and youth reports were moderately correlated in females. However, the correlations were notably weaker at baseline (r = 0.57) and the last time point (r = 0.62). In males, parent and youth reports were less correlated across all time points in comparison to females, but especially weak at baseline (r = 0.24) and the second time point (r = 0.41). Overall, youth were more likely to endorse “I do not know” or “Refuse to Answer” at the two earlier time points. The data loss due to this made the derivation of pubertal timing and tempo from the youth report much less stable. As a sensitivity analysis, we ran our models using parent-only and a combined version (parent report from the first two time points and youth report from the last three time points). The results in predicting structural brain development were highly similar. However, the parent-only derivation model had better fit indices than that of the combined version (see the Supplementary Materials for model fit indices and the combined version results from the focal models). Therefore, we used the parent-only report as our timing and tempo derivation and report these results in the main text.

#### 2.4.3 Models

We used generalized additive mixed models (GAMMs) to assess our questions, utilizing *mgcv* (v1.9–1) in R version 4.0.2. Models were run separately for males and females. Our outcome variable was a region's volume, cortical thickness, or surface area (see the *Brain* section above for more details). We ran three models for timing and three models for tempo, resulting in six model types for each outcome. We used a False Discovery Rate of 5 % to account for multiple comparisons across brain regions within each brain metric (i.e., thickness, surface, volume). All models included a smoothed age (standardized) term. A smoothed variable in GAMM is a term that allows the effect of the predictor to be flexible and nonlinear. We specified the basis function of age in our models to be a cubic regression spline with shrinkage, using 7 knots (initially set to 5, but model performance metrics using *gam.check* suggested increasing the number of knots). Pubertal timing and tempo were entered as standardized linear predictors to ensure model convergence.

Model 1: Brain ∼ age

Model 2a: Brain ∼ age + pubertal timing

Model 3a: Brain ∼ age + pubertal timing + age*pubertal timing

Model 2b: Brain ∼ age + pubertal tempo

Model 3b: Brain ∼ age + pubertal tempo + age*pubertal tempo

All models were nested within an individual, including site ID as dummy-coded control variables (site ID one as the reference). Model 1 was then compared to Model 2a or b, Model 2a or b to Model 3a or b, and Model 1 to Model 3a or b using *anova* from the *mgcv* package in R (v4.0.2). Pubertal timing and tempo models were not statistically compared because they were not nested, meaning their respective model complexities cannot be compared. The function *gam.check* was used to assess model fit. The code for these analyses is available here: https://github.com/clarefmccann/abcd-pub-smri-publication. See Supplementary Materials for all model comparison statistics. A significant improvement in model fit means adding the new term, in this case either a main effect of puberty (Model 2) or an interaction term between puberty and age (Model 3), improves the statistical model’s ability to predict the development of a region (a.k.a. this term contributes to the variance observed).

A significant effect of pubertal timing can be interpreted as a difference in brain development attributed to an individual’s pubertal timing, above and beyond age. For example, a positive effect of pubertal timing on cortical surface area would indicate that, on average, individuals with a younger age at Tanner Stage 3 (or earlier timing) exhibit less cortical surface area than those with older ages at Tanner Stage 3. A positive effect of pubertal tempo on cortical surface area, for example, indicates that, on average, individuals with faster pubertal tempo exhibit larger cortical surface area than same-aged, same-sex peers. A significant interaction term would suggest that individual differences in pubertal timing or tempo are associated with varying rates (either faster or slower) of cortical or subcortical development over age.

## 3 Results

### 3.1 Effects of age on brain development

See Supplementary Materials for results from Model 1, which predicted structural brain development while accounting for age and control variables, but not puberty. Effects were only considered significant after FDR correction.

### 3.2 Pubertal timing

**Fig. 2:**
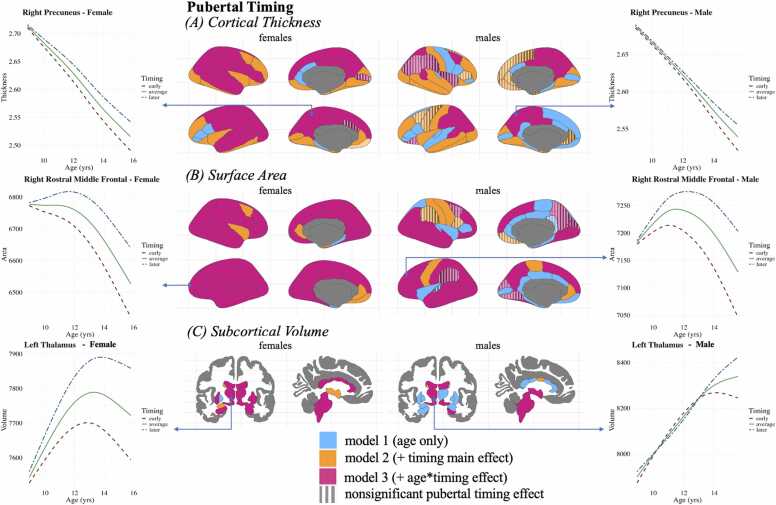
Best-fitting model among pubertal timing models for each region. Light blue regions were best predicted by model 1 (age only), orange regions were best predicted by model 2a (main effect of pubertal timing), and pink regions were best predicted by model 3a (interaction effect of age and pubertal timing). Regions patterned with stripes did not have a significant additional term (i.e., even though the pubertal timing main effect model best predicted a region, the pubertal timing main effect estimate was not significant). Examples of developmental trajectories are provided, with the red dashed line indicating earlier pubertal timing, the solid green line indicating average pubertal timing, and the blue dashed and dotted line indicating later pubertal timing. These patterns of development are primarily representative of the other significantly predicted regions, with slight variation in the shape of development. Refer to the OSF for the present project for visualizations of all significant effects (https://osf.io/mgjyh/files/sdy6k).

See Fig. 2 for a map of regions best predicted by each model. Effects were only considered significant after FDR correction.

#### 3.2.1 Main effect of pubertal timing

*Cortical Thickness.* Model 2a involved adding the pubertal timing main effect term to the simpler Model 1, which contained only age and control variables (see Supplementary Materials for Model 1 results). Adding pubertal timing significantly improved the model fit for the development of thickness in 33.8 % of the regions in females. These regions included the bilateral insular cortex, right medial orbitofrontal cortex, and the right caudal and bilateral rostral middle frontal gyri. Model 2a was the best fit for the right entorhinal and left medial orbitofrontal cortices, but pubertal timing did not significantly predict cortical thickness in this region. Pubertal timing was a significant predictor of cortical thickness in all other regions for which Model 2a was the best-fitting model (in females). For regions that were significantly predicted by pubertal timing, on average, females with earlier pubertal timing had ∼0.01 mm (SD = 0.003) less cortical thickness than their same-aged, same-sex peers.

The pubertal timing main effect model (Model 2a) significantly improved the model fit for the development of thickness in 29.4 % of the regions in males. These regions included the left insular cortex, right lateral orbitofrontal cortex, and the bilateral rostral middle frontal gyrus. The right entorhinal, right medial orbitofrontal, left precuneus, bilateral rostral cingulate cortices, and right superior frontal and right caudal middle frontal gyri were not significantly predicted by pubertal timing, despite Model 2 being the best-fitting region for these models. In regions that were significantly predicted by pubertal timing, on average, males with earlier pubertal timing have ∼0.008 mm (SD = 0.002) less cortical thickness than their same-aged, same-sex peers. All age effects remained significant (Mean EDF = 3.41, SD EDF = 0.53 in females; Mean EDF = 3.14, SD EDF = 0.61 in males) after adding the pubertal timing main effect term, excluding the bilateral entorhinal cortex in females.

*Surface Area.* Adding pubertal timing (Model 2a) significantly improved the model fit for surface area in 11.8 % of the regions in females. Females with earlier pubertal timing exhibit ∼12.15 mm² (SD = 11.78) less surface area on average compared to same-aged, same-sex peers in regions such as the right caudal middle frontal gyrus, right insular cortex, and bilateral rostral anterior cingulate cortex.

Adding pubertal timing (Model 2a) improved the model fit for the development of surface area in 13.2 % of the regions in males, with six regions showing significant effects (including the precentral gyrus and the left rostral anterior cingulate cortex), having a mean effect size of 19.70 mm² (SD = 14.35). Interestingly, the right frontal pole was significantly predicted by pubertal timing but not age. On average, males with earlier pubertal timing had less surface area than their same-aged, same-sex peers. All age effects remained significant (Mean EDF = 2.25, SD EDF = 0.38 in females; Mean EDF = 2.22, SD EDF = 0.34 in males) after adding the pubertal timing main effect term, other than the right frontal pole in females.

*Subcortical Volume.* Model 2a significantly improved the model fit of 16.6 % of the brain’s subcortical volume in females relative to Model 1 and 3a. The effect sizes of pubertal timing had a mean difference of 0.95 mm³ (SD = 15.58) in volume on average, although the effects varied in direction. Pubertal timing exhibited a negative relationship with the third ventricle, cerebrospinal fluid, and left inferior lateral ventricle, such that females with earlier pubertal timing exhibited greater volume in these regions on average. Positive effects were observed between pubertal timing and the right nucleus accumbens, left amygdala, and left caudate, indicating that females who exhibited earlier pubertal timing had, on average, less volume in these regions. All age effects remained significant for regions best explained by Model 2a (Mean EDF = 2.84, SD EDF = 0.46).

Model 2a significantly improved the prediction of the bilateral nucleus accumbens, left caudate, and the central corpus callosum in males relative to Model 1. However, the left caudate was not significantly predicted by pubertal timing or age. Males with earlier pubertal timing exhibited 6.95 mm³ more volume on average in the central corpus callosum. Males with earlier pubertal timing exhibited 5.75 mm³ less volume on average in the bilateral nucleus accumbens area. All age effects remained significant (Mean EDF = 2.46, SD EDF = 0.45).

#### 3.2.2 Age x pubertal timing

*Cortical Thickness.* Including the interaction between age and pubertal timing (Model 3a) significantly improved model fit for 52.9 % of the cortical regions in females, suggesting that cortical thickness in these regions is best predicted by a model where age effects vary as a function of pubertal timing. Regions best predicted by Model 3 included the bilateral precuneus cortex, right middle temporal, and the right caudal middle frontal gyri. All regions were significantly predicted by the interaction between age and pubertal timing (Mean EDF = 1.05, SD EDF = 0.14), other than the left caudal anterior-cingulate and right pericalcarine cortices (although they did have significant pubertal timing main effects; b = 0.008 mm, 0.006 mm, respectively). Participants with earlier pubertal timing exhibited accelerated rates of cortical thinning relative to their peers in all regions. All age effects remained significant (Mean EDF = 3.68, SD EDF = 0.34).

Cortical thickness in males was best predicted by Model 3a in 26.47 % of regions, such as the bilateral superior parietal, bilateral precuneus, and right isthmus cingulate cortices. All interaction effects were significant (Mean EDF = 1.21, SD EDF = 0.34) except the right inferior parietal, right insular, left lateral orbitofrontal cortices, and right supramarginal gyrus (although they did have significant pubertal timing main effects; b = 0.007 mm, 0.009 mm, 0.004 mm, 0.008 mm, respectively). Participants with earlier pubertal timing exhibited accelerated reductions in cortical thickness. All age effects remained significant (Mean EDF = 3.51, SD EDF = 0.29).

*Surface Area.* Adding the interaction term between age and pubertal timing (Model 3a) significantly improved the model fit, predicting surface area development in 83.82 % of 68 regions for females and 52.94 % of 68 regions in males. In females, this pattern was observed in the vast majority of the cortex, with exceptions in the medial orbitofrontal cortex and portions of the temporal lobe. In females, the surface area development of all regions was significantly predicted by the interaction between age and pubertal timing (Mean EDF = 1.14, SD EDF = 0.17), except for the left temporal pole. However, it did have a significant main effect of pubertal timing (b = 2.39 mm^2^).

In males, all regions other than the bilateral cuneus and right precuneus cortices, and left lingual, right caudal middle frontal, and left supramarginal gyri were significantly predicted by the interaction between age and pubertal timing (Mean EDF = 1.21, SD EDF = 0.47). However, the right precuneus cortex and right caudal middle frontal and left supramarginal gyri exhibited significant main effects of pubertal timing (b = ∼38.75 mm^2^, SD = 3.06). Participants with earlier pubertal timing exhibited accelerated rates of surface area development relative to their peers in all regions. All age effects remained significant (Mean EDF = 3.17, SD EDF = 0.30 in females; Mean EDF = 2.76, SD EDF = 0.25 in males), other than the left temporal pole in females and the bilateral cuneus cortex in males.

*Subcortical Volume.* Adding the interaction term between age and pubertal timing (Model 3a) significantly improved the model fit, predicting volumetric development in 70 % of 40 regions in females (including the right amygdala, right caudate, bilateral hippocampus, bilateral putamen, and right pallidum) and 40 % of 40 areas (including the right hippocampus, left thalamus, and bilateral ventral diechepalon) in males. For females, the volumetric development in all regions best predicted by Model 3a showed significant interaction effects (Mean EDF = 1.77, SD EDF = 0.85). For males, the volumetric development in all regions best predicted by Model 3a showed significant interaction effects (Mean EDF = 2.11, SD EDF = 0.61), excluding the right caudate. For significant effects, participants with earlier pubertal timing exhibited accelerated rates of volumetric development relative to their peers in all regions. All age effects remained significant (Mean EDF = 3.43, SD EDF = 0.57 in females; Mean EDF = 3.83, SD EDF = 0.77 in males).

### 3.3 Pubertal tempo

**Fig. 3:**
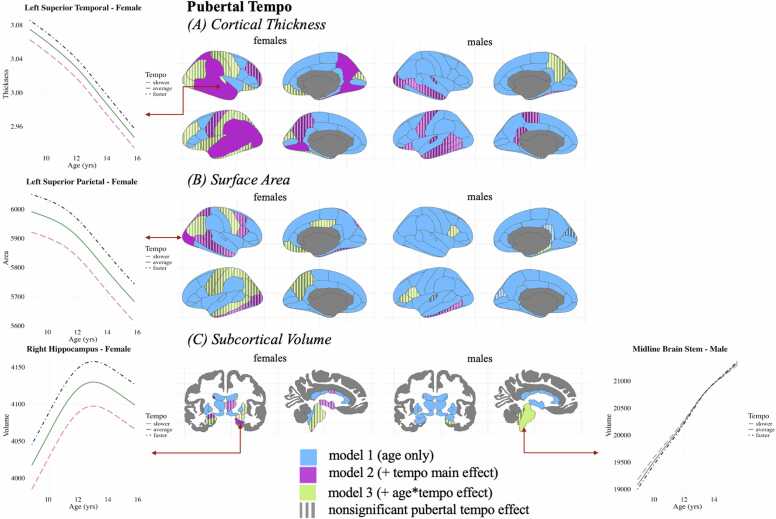
Best-fitting model among pubertal tempo models for each region. Bright blue regions were best predicted by model 1 (age only), bright purple regions were best predicted by model 2b (main effect of pubertal tempo), and bright green regions were best predicted by model 3b (interaction effect of age and pubertal tempo). Regions patterned with stripes did not have a significant additional term (i.e., even though the pubertal tempo main effect model best predicted a region, the pubertal tempo main effect estimate was not significant). Examples of developmental trajectories are provided, with the pink dashed line indicating a slower pubertal tempo, the solid green line indicating an average pubertal tempo, and the dark blue dashed and dotted line indicating a faster pubertal tempo. Refer to the OSF for the present project for visualizations of all significant effects (https://osf.io/mgjyh/files/sdy6k).

See Fig. 3 for a map of regions best predicted by each model. Effects were only considered significant after FDR correction.

#### 3.3.1 Main effect of pubertal tempo

*Cortical Thickness.* Model 2b involved adding the pubertal tempo main effect term to the simpler Model 1, which contained only age and control variables (see Supplementary Materials for Model 1 results). Model 2b significantly improved the fit for the development of cortical thickness in 33.8 % of 68 regions in females (including the right precuneus cortex and bilateral middle temporal gyrus) and in 17.6 % of 68 regions in males (including the left lateral orbitofrontal cortex and left inferior temporal gyrus). However, none of the regions were significantly predicted by the main effect of pubertal tempo in males.

The development of cortical thickness in most regions best predicted by Model 2b showed significant positive main effects of pubertal tempo in females, meaning that those with a faster tempo exhibited thicker cortices on average in these regions. On average, females with a faster pubertal tempo have ∼0.009 mm (SD = 0.002) thicker cortices than their slower tempo same-aged, same-sex peers. The left precuneus cortex, left precentral gyrus, right rostral middle frontal gyrus, and right temporal pole were not significantly predicted by pubertal tempo. All age effects associated with cortical thickness development remained significant after accounting for pubertal tempo in regions that were best predicted by Model 2b (Mean EDF = 3.58, SD EDF = 0.57 in females; Mean EDF = 3.12, SD EDF = 0.47 in males), other than the left temporal pole in females.

*Surface Area.* The pubertal tempo main effect model (Model 2b) significantly improved the fit for the development of 13.2 % of 68 regions in females and the left inferior temporal gyrus in males. In females, only the right lateral occipital cortex was significantly predicted by the main effect of pubertal tempo. In males, the left inferior temporal gyrus was not significantly predicted by the main effect of pubertal tempo. Females with a faster pubertal tempo have ∼37.89 mm^2^ more surface area than their slower tempo same-aged, same-sex peers in the right lateral occipital cortex. All age effects remained significant after accounting for pubertal tempo in regions that were best predicted by Model 2b (Mean EDF = 3.19, SD EDF = 0.24 in females; EDF = 2.75 in males).

*Subcortical Volume.* The pubertal tempo main effect model (Model 2b) significantly improved the model fit for 14.7 % of the 40 regions in females and none in males. Only the bilateral cerebellum cortex and right hippocampus were significantly predicted by pubertal tempo in females. Females with faster pubertal tempo exhibit ∼406.73 mm^3^ (SD = 5.17) more volume in the bilateral cerebellum cortex and 24.28 mm^3^ more volume in the right hippocampus than slower tempo same-aged, same-sex peers. All age effects for regions’ volumetric development, best predicted by Model 2b, remained significant (Mean EDF = 3.53, SD EDF = 0.59).

#### 3.3.2 Age x pubertal tempo

*Cortical Thickness.* Including the interaction between age and pubertal tempo (Model 3b) significantly improved the prediction of cortical thickness development in 23.5 % of the regions in females and the right precuneus in males, although none of the interaction terms significantly predicted any regions in females or males. However, all but three regions (the left insular cortex, right lateral orbitofrontal cortex, and right precentral gyrus) exhibited significant pubertal tempo main effects in females. On average, females with a faster pubertal tempo exhibited ∼0.007 mm (SD = 0.002) thicker cortices than their same-aged, same-sex peers with a slower pubertal tempo. All age effects for regions’ cortical thickness development best predicted by Model 3b remained significant (Mean EDF = 3.56, SD EDF = 0.34 in females; EDF = 3.91 in males).

*Surface Area.* Adding the interaction term between age and pubertal tempo (Model 3b) significantly improved the model fit in predicting surface area development, with improvements observed in 16.17 % of 68 regions for females and 5.8 % of 68 regions in males. No interaction terms were significant for males or females. Although the left superior parietal cortex in females exhibited a significant main effect of pubertal tempo, such that females with faster tempo exhibited 52.29 mm^2^ more surface area compared to same-aged, same-sex females with slower tempo. All age effects for regions’ cortical thickness development best predicted by Model 3b remained significant (Mean EDF = 3.01, SD EDF = 0.55 in females; Mean EDF = 2.67, SD EDF = 0.26 in males).

*Subcortical Volume.* Adding the interaction term between age and pubertal tempo (Model 3b) significantly improved the model fit, predicting the volumetric development of 12.5 % of regions in females and 15 % of regions in males. No interaction terms were significant in females. The brain stem (EDF = 1.11), right cerebellum white matter (EDF = 1.05), and the bilateral cerebellum cortex (Mean EDF = 1.05, SD EDF = 0.07) all exhibited significant interaction effects in males, indicating that age-related volumetric development differs by pubertal tempo in these regions. In those regions, males with a faster pubertal tempo exhibited steeper increases in volume compared to their slower-tempo same-aged, same-sex peers. All age effects for regions’ volume development best predicted by Model 3b remained significant (Mean EDF = 3.46, SD EDF = 0.61 in females; Mean EDF = 4.06, SD EDF = 0.61 in males).

## 4 Discussion

The study investigated the role of variability in pubertal development in predicting structural brain development during adolescence. Specifically, we examined the roles of chronological age, pubertal timing (i.e., an individual’s deviation from the sample-derived estimate of age at Tanner Stage 3 compared to same-aged, same-sex peers), and tempo (i.e., deviation in the speed of progression) to identify sources of variability in structural brain development during adolescence. We found that pubertal timing and tempo significantly contributed to the development of cortical thickness, surface area, and subcortical volume during adolescence.

### 4.1 Main effects of pubertal timing as a predictor of neurodevelopment

Including pubertal timing as a predictor improved model fit for cortical thickness predominantly in females, but smaller regions of significance were also evident in males. Earlier pubertal timing was associated with overall thinner cortices, consistent with accelerated maturation. These effects were strongest in frontal, temporal, and occipital cortices in females and in parietal regions in males. The fewer significant effects in males likely reflect their later pubertal onset during this age range rather than an absence of association.

Several biological processes underlie cortical thickness, including synaptic pruning, dendritic branching, and intracortical myelination. Animal research in rodents reveals that sex steroids and pubertal development influence the number of synapses, dendritic branching, and myelination processes, with diverse region- and sex-specific effects (discussed further in Herting and Sowell, 2017). Other work suggests that puberty may indirectly influence brain development through changes in the social environment linked to sex steroids and physical changes associated with puberty (Blakemore et al., 2010).

These findings replicate prior research in the ABCD Study, which found that individuals with earlier pubertal timing exhibit thinner cortices on average in temporal, frontal, occipital, and parietal regions (MacSweeney et al., 2023, Wiglesworth et al., 2023). However, our work also extends these prior findings by examining changes in structural development across three time points of longitudinal data. Three time points allow us to assess the effect of pubertal timing at later ages. Effects of pubertal timing may change across adolescence. For example, a null finding at one point in development can emerge or reverse at another.

Our findings in males contrast with findings from MacSweeney et al. (2023) and Beck et al. (2023), who found no effect of pubertal timing on male cortical thickness. However, we also replicate previous findings, which found effects of pubertal timing on cortical thickness in males (Holm et al., 2023, Whitmore et al., 2023, Wiglesworth et al., 2023), although Wiglesworth et al. (2023) found that these effects were stronger in females. Our metric of pubertal timing differs from prior work in that we derived an individual’s deviation from the sample-derived age at Tanner Stage 3. This approach enhances the interpretability of our findings by anchoring timing to a specific developmental milestone. Thus, this method may capture a more precise and meaningful index of pubertal timing rather than a general measure of timing. Furthermore, since we used three time points instead of two, our analysis also includes pubertal timing effects at later ages. These contrasting results suggest that the strength of the relationship between pubertal timing and cortical thickness development may differ in later ages.

Earlier pubertal timing was associated with a smaller cortical surface area in both males and females. Including pubertal timing as a predictor improved model fit and significantly explained variation in the surface area of frontal and limbic regions in females, with slightly more widespread effects in males. These sex differences could reflect differences in the developmental timing of puberty (similar to the cortical thinning results). Widespread differences of cortical surface area in females may occur earlier than in males, and thus, only later-developing brain regions were observed as having differences in cortical surface area during the developmental window examined here. The development of cortical surface area is shaped partly by distinct biological mechanisms, which most likely explain the divergence in neural metrics. This pattern contrasts with MacSweeney et al. (2023), who found no effect of pubertal timing on surface area, but aligns with Curtis et al. (2024), who found that hormonal pubertal timing was a strong predictor of cortical surface area, and with Beck et al. (2023), who observed larger decreases surface area in males and females with earlier timing.

Including pubertal timing significantly improved the model fit for subcortical volume in males and females. In females, earlier pubertal timing was linked to greater ventricular and CSF volume and smaller volume in the right nucleus accumbens, left amygdala, and left caudate. In males, earlier pubertal timing was associated with a smaller volume of the bilateral nucleus accumbens and a larger volume of the central corpus callosum. Model fit for the volume of the left caudate also improved, although the effect itself was nonsignificant. These results are consistent with prior findings that pubertal timing influences subcortical volume in both males and females (Goddings et al., 2014, Wierenga et al., 2018). However, this finding differs from earlier analyses in the ABCD Study using only two time points, which found no relationship between amygdala volume and pubertal timing (Thijssen et al., 2020, Thijssen et al., 2022), suggesting that the effect of pubertal timing may differ in strength at later ages.

### 4.2 Age x pubertal timing interactions predict most features of structural brain development

Widespread age-by-pubertal timing interactions were observed across numerous brain regions for cortical and subcortical development. This suggests that the rate of change in brain development from ages 9–15 years differed depending on pubertal timing in the frontal, occipital, temporal, parietal, and limbic cortices. Female brain development during this period appears to be more sensitive to the interaction between age and pubertal timing, as fewer regions showed significant effects in males.

Regions showing significant interactions displayed patterns indicative of accelerated neural maturation among adolescents with earlier pubertal timing. In these regions, neither age nor pubertal timing alone adequately explained variability in structural brain development, emphasizing the dynamic nature of their interplay. Anchoring timing to a specific developmental milestone, such as deviation from the sample-derived age at Tanner Stage 3, may therefore improve the predictive value of neurodevelopmental models and refine the timing of intervention or prevention efforts. For instance, brain regions sensitive to socioemotional learning may reach their most plastic point at an earlier age in adolescents who reach Tanner Stage 3 earlier than their peers, making them more vulnerable to environmental influences at younger ages and less malleable later on.

### 4.3 Pubertal tempo as a predictor of brain development across adolescence

Notably, our study investigated the role of pubertal tempo in structural brain development, a topic that has been rarely explored due to the need for longitudinal data. Pubertal tempo may influence neural maturation by determining the duration of exposure to specific hormonal processes, with slower or faster tempo potentially relating to prolonged or condensed periods of neuroendocrine activity (Herting et al., 2015). It is unknown how pubertal tempo impacts sensitivity to one’s broader socioemotional context, if at all, and is a topic worthy of future study.

Evidence for pubertal tempo is sparse, with existing studies typically relying on interval-based change scores that cannot capture an individual’s rate of progression in the context of the developmental process of puberty. Using these approximate measures, findings indicate that larger increases in pubertal stage between two time points are associated with less thinning in the superior frontal cortex and larger decreases in an advanced composite of neural maturation (Herting et al., 2015, Holm et al., 2023). One study found that faster tempo specifically related to subcortical and cortical development in male individuals (Vijayakumar et al., 2021, Vijayakumar et al., 2021).

While we did not replicate the significant effects found between pubertal tempo and rate of development in cortical thickness or surface area, we did find that age-related trajectories of volume in the brain stem, the cerebellum cortex, and cerebellum white matter varied by pubertal tempo in males. Further, our main effect findings of pubertal timing being related to greater volume in the right hippocampus on average align with Vijayakumar et al. (2021a) depiction of the developmental trajectory of the right hippocampus, such that individuals with faster pubertal tempo exhibited greater volume across adolescence. They found this only in males, while we saw this in females; however, they derived tempo from a linear mixed model due to a smaller number of time points, potentially missing critical developmental nuances characterizing pubertal tempo. In the present paper, the right hippocampus was best predicted by the pubertal tempo interaction model, but the term itself was not significant. The present paper differs from these previous studies in that we use a larger sample with more time points, which extend beyond the age of 14. Therefore, we have more individuals at later stages of puberty and use a specific developmental milestone (Tanner Stage 3) as the anchor for tempo.

Compared to pubertal timing, pubertal tempo significantly improved the model fit and significantly predicted development in fewer regions for both females and males. Pubertal tempo seemed most relevant to cortical thickness in females, with the parietal and occipital regions exhibiting significant main effects, indicating that those with faster tempo had thicker cortices in these regions on average. Across all brain metrics and sexes, the pubertal tempo interaction model improved the model fit for some regions; however, the interaction term itself was not significant. Within some of these models, the pubertal tempo main effect was significant, suggesting that adding the interaction term improved the predictive utility of the model; however, it does not significantly predict the development of these regions. While pubertal tempo’s effect may not vary much by age, it may help explain additional variance in average differences in brain structure during adolescence when used as a complementary predictor to pubertal timing.

Pubertal timing and tempo were moderately correlated (r = 0.35 in females, r = -0.32 in males), but not perfectly, indicating that they capture unique yet related characteristics of pubertal development. Although we did not statistically compare our pubertal timing and tempo models, we observed a few differences in the results. For example, in males, the cortical thickness of the left isthmus cingulate cortex and bilateral transverse temporal cortex, as well as the surface area of the right parahippocampal gyrus, were not best predicted by pubertal timing models, but were best predicted by pubertal tempo models. These findings suggest that pubertal tempo may explain variance in the development of different regions than pubertal timing, warranting future research to disentangle the unique effects of these two maturational markers.

### 4.4 Limitations and future directions

The present study has numerous strengths, including its longitudinal design and large sample size. At the same time, like all studies, there are several limitations to note that can be addressed in future designs. First, these findings relied exclusively on reports of pubertal development, which reflect perceptions of physical changes rather than puberty as a comprehensive process. Reports are also collected from parents and participants in the larger ABCD Study, which trade off the degree of reliability over time. The present study relied solely on parent reports, but future studies should consider more complex measurement modeling to parse the shared and unique variance among the given reports. Second, due to the age range of our sample, females were generally further along in their pubertal development than males. Our results primarily capture the middle to late stages of puberty for females, which means we may have missed critical developmental nuances in structural brain development that occur closer to the actual onset of puberty. Third, although our approach offers novel insight into how specific aspects of pubertal development influence structural brain changes, our estimates were derived from a statistical model and not from direct reports of age at Tanner Stage 3, which may result in some degree of extrapolation for individuals whose data did not include this specific transition point. However, the modeling approach used to estimate pubertal timing and tempo is designed to interpolate and project developmental trajectories both backward and forward from available observations, allowing for reasonable approximations even in the absence of direct reports. Fourth, pubertal timing and tempo were entered as linear predictors and in separate models to ensure model convergence. In future releases of the ABCD Study® data, with the remainder of year four follow-up and into year six, more complex nonlinear models, including more interaction terms, may be more stable. Finally, although the ABCD Study® sample is collected across 21 locations and is relatively diverse, the sample in the present study is not nationally representative. In our specific subset sample, Black and Hispanic youth were more likely to be excluded from the analyses. Future work should interrogate the reasons for the discrepancy in exclusion likelihood and perform similar analyses in samples with representative numbers of Black and Hispanic youth.

## 5 Conclusion

Our results suggest that puberty contributes significantly to the variance in neurodevelopment during adolescence, not accounted for by chronological age. Individuals with earlier timing tend to exhibit accelerated normative developmental trajectories, while those with faster tempos tend to have thicker cortices, greater surface area, and higher volume on average. We also found differences in the effects of pubertal timing and tempo on brain development between males and females, specifically in the development of cortical thickness. However, this could in part be due to the age range of our sample. Given that pubertal timing and tempo predicted cortical and subcortical development in several regions implicated in socioemotional learning, this may indicate a mechanism by which pubertal processes coordinate brain development in general or perhaps how it is shaped by social experiences during adolescent development.

## Funding Sources

This work was supported by the 10.13039/100000002National Institutes of Health #R21HD108751 (PIs: J.A. Silvers, N. Eisenberger, M. Lieberman) and the 10.13039/100000071National Institute of Child Health and Human Development #2T32HD091059-06A1 (PIs: A.J. Fuligni, A. Galván, M. Dapretto).

Data used in the preparation of this article were obtained from the Adolescent Brain Cognitive DevelopmentSM (ABCD) Study (https://abcdstudy.org), held in the NIMH Data Archive (NDA). This is a multisite, longitudinal study designed to recruit more than 10,000 children age 9–10 and follow them over 10 years into early adulthood. The ABCD Study® is supported by the National Institutes of Health and additional federal partners under award numbers U01DA041048, U01DA050989, U01DA051016, U01DA041022, U01DA051018, U01DA051037, U01DA050987, U01DA041174, U01DA041106, U01DA041117, U01DA041028, U01DA041134, U01DA050988, U01DA051039, U01DA041156, U01DA041025, U01DA041120, U01DA051038, U01DA041148, U01DA041093, U01DA041089, U24DA041123, U24DA041147. A full list of supporters is available at https://abcdstudy.org/federal-partners.html. A listing of participating sites and a complete listing of the study investigators can be found at https://abcdstudy.org/consortium_members/. ABCD consortium investigators designed and implemented the study and/or provided data but did not necessarily participate in the analysis or writing of this report. This manuscript reflects the views of the authors and may not reflect the opinions or views of the NIH or ABCD consortium investigators.

## CRediT authorship contribution statement

**Theresa W. Cheng:** Writing – review & editing, Conceptualization. **Kathryn L. Mills:** Writing – review & editing, Conceptualization. **Jennifer A. Silvers:** Writing – original draft, Supervision, Conceptualization. **Clare F. McCann:** Writing – original draft, Project administration, Methodology, Investigation, Formal analysis, Conceptualization.

## Declaration of Competing Interest

The authors declare that they have no known competing financial interests or personal relationships that could have appeared to influence the work reported in this paper.

## Data Availability

I have attached the link to the data in the data statement file.
