# Supplementary material for: Pubertal timing and tempo differentially influence cortical and subcortical maturation in adolescence
Source: Dev Cogn Neurosci. 2025 Dec 12;77:101657. doi: 10.1016/j.dcn.2025.101657 (PMC12767848; doi:10.1016/j.dcn.2025.101657)
Supplement: Supplementary file 1 — Supplementary material [file mmc1.docx]


**Figure 1. Exclusion Flowchart of the Present Study.** Note: 3 individuals were removed due to being intersex. 129 observations (not participants) were removed during the longCombat step of the present study because they were collected on scanners with too few observations to reliably harmonize the data.
