# Supplementary material for: Pubertal timing and tempo differentially influence cortical and subcortical maturation in adolescence
Source: Dev Cogn Neurosci. 2025 Dec 12;77:101657. doi: 10.1016/j.dcn.2025.101657 (PMC12767848; doi:10.1016/j.dcn.2025.101657)
Supplement: Supplementary file 2 — Supplementary material [file mmc2.docx]

**Declaration of interests**
 
☒ The authors declare that they have no known competing financial interests or personal relationships that could have appeared to influence the work reported in this paper.
 
☐ The authors declare the following financial interests/personal relationships which may be considered as potential competing interests:
